# Annual acknowledgement of manuscript reviewers

**DOI:** 10.1186/1472-6750-13-20

**Published:** 2013-04-04

**Authors:** Christopher Morrey

**Affiliations:** 1 BioMed Central, Floor 6, 236 Gray's Inn Road, London WC1X 8HB, UK

## Abstract

**Contributing reviewers:**

The editors of BMC Biotechnology would like to thank all of our reviewers who have contributed to the journal in Volume 12 (2012).

## 

Jose Manuel Ageitos

Spain

Ishtiaq Ahmed

Pakistan

Cecile Albenne

France

Jennifer Alcaino

Chile

Andrea Alford

USA

Graca Almeida-Porada

USA

Mikael Rordam Andersen

Denmark

Maciek Antoniewicz

USA

Miguel Aranda

Spain

Christopher Arevalos

USA

William John Armitage

UK

Anjon Audhya

USA

Bernhard Auer

Austria

Karen Avraham

Israel

Daris Balciunas

USA

Jaouadi Bassem

Tunisia

Heinz Baumann

USA

Helmut Bäumlein

Germany

Edward A. Bayer

Israel

Frederic Beaudoin

UK

Michele Bellucci

Italy

William Bentley

USA

R Howard Berg

USA

Imre Berger

France

Gonçalo Bernardes

Switzerland

Subhash Bhore

Malaysia

Lijun Bi

China

Peggy Biga

USA

Jose M. Blanca

Spain

Lars Mathias Blank

Germany

Maria Jose Bonete

Spain

Dominique Böttcher

Germany

Marc Boutry

Belgium

Claudio Brancolini

Italy

David Brand

USA

Adriano Brandelli

Brazil

Charlotte Brandt Sørensen

Denmark

Catarina Brito

Portugal

David Brummell

New Zealand

Leif Bulow

Sweden

Michael Butler

Canada

Lisa Cabrita

UK

Kaiyong Cai

China

Johanna Camara

USA

Silvia Camperi

Argentina

Melanie Carless

USA

Elise Champion

Denmark

Wen-Tsan Chang

Taiwan

Janet Chantler

Canada

Chunxian Chen

USA

Jong Hyun Choi

South Korea

Patrick Cirino

USA

Manon Cox

USA

Olivier Danos

France

Marc-Andre D'Aoust

Canada

Atze Das

Netherlands

Paulo De Boer

Netherlands

Vincenzo De Luca

Canada

Nicole Déglon

Switzerland

Francisco Javier Deive Herva

Spain

Amandine Delteil

France

Ann Depicker

Belgium

Ram Devireddy

USA

Michael K Deyholos

Canada

Ursula Dietrich

Germany

Lucilia Domingues

Portugal

Francisco Dos Santos

Portugal

Brian Driscoll

Canada

Liangcheng Du

USA

Colin Eady

New Zealand

Jerry Eichler

Israel

Vincent Eijsink

Norway

David Engelke

USA

Wolfgang Ernst

Austria

Dominic Esposito

USA

Jose Estevez

Argentina

Mary Farach-Carson

USA

Boris Fehse

Germany

Xingjun Feng

China

Carmen Fenoll

Spain

Tiago Fernandes

Portugal

Frederico Ferreira

Portugal

Alfred Fisher

USA

Elizabeth Fitzpatrick

USA

Tom Flanagan

Ireland

Guillermina Forno

Argentina

Marco Fraaije

Netherlands

Octavio Franco

Brazil

Todd French

USA

Douglas Fudge

Canada

Ying Sing Fung

Hong Kong

Vijay Gadkar

Canada

Igor Galaev

Netherlands

Melanie Galla

Germany

Anne Galy

France

Lucia Gardossi

Italy

Mesa Garriga

Cuba

Vivek Kumar Garripelli

USA

Sudeep George

USA

Pallavi Ghosh

USA

Luiz Antonio Gioielli

Brazil

Ashok Giri

India

Tor Gjøen

Norway

Andrew Gleave

New Zealand

Daniel Gorelick

USA

Johann Gorgens

UK

Allison Groseth

USA

Karl Gruber

Austria

Georg Guebitz

Austria

Munishwar Gupta

India

Rani Gupta

India

Ramneek Gupta

Denmark

Melissa Gutarra

Brazil

Peter Leon Hagedoorn

Netherlands

Claudia Hagedorn

Germany

Donny Hanjaya-Putra

USA

Brendon Hanson

Singapore

Israel Hanukoglu

Israel

Tadashi Hatanaka

Japan

Mingyue He

UK

Jean-Michel Heraud

Madagascar

Holly Hogrefe

USA

Hue Tran Hornig-Do

UK

Huey-Chun Huang

Taiwan

Georg Hubmann

Belgium

Michael Hust

Germany

Larissa Ivanova

Russian Federation

Zoltan Ivics

Germany

Hiro-Oki Iwakawa

Japan

Yves Jacob

USA

Narendra Jawali

India

Jihong Jiang

China

Peng Jiao

USA

Sha Jin

USA

Djuro Josic

Croatia

Abul Kalam

Saudi Arabia

Vipin Kalia

India

Héla Kallel

Tunisia

Jeffrey Karp

USA

Brian Kaspar

USA

Tatsuya Kato

Japan

Masahiro Kawahara

Japan

Hideya Kawaji

Japan

Koichi Kawakami

Japan

Joanne Keenan

Ireland

Yeon-Gu Kim

South Korea

Yong Hwan Kim

South Korea

Kyoung Heon Kim

South Korea

Robin Kimmel

Austria

Linda King

UK

Ralph Kirby

Taiwan

David Kirk

USA

Harald Kirsebom

Sweden

Michihiko Kobayashi

Japan

Daisuke Kohda

Japan

Roland Kontermann

Germany

Giampiero La Rocca

Italy

Cristiano Lacorte

Brazil

Geoff Lane

New Zealand

Nigel Larsen

New Zealand

Kent Leach

USA

Charles Lee

USA

Gyun Min Lee

South Korea

Hans Leemhuis

Netherlands

Matic Legisa

Slovenia

Xingfang Li

Canada

Yulin Li

Portugal

Wan-Ju Li

USA

Patrick Limbach

USA

Maria Limberis

USA

Min Lin

Canada

Ying Lin

China

Andreas James Link

USA

Hwei San Loh

Malaysia

George Lomonossoff

UK

Gang Lu

China

Hong Luo

USA

Georgina Macintyre

Canada

Richard Colin Macknight

New Zealand

Catarina Madeira

Portugal

Richard Helmut Maier

Austria

Pal Maliga

USA

Mickael Malnoy

Italy

Rita Malpique

Spain

Michael Martin

Germany

Ludmila Martinkova

Czech Republic

Kazunori Matsuda

Japan

Ken Matsuoka

Japan

Sean Mayes

UK

Stephen Mayfield

USA

Karen McDonald

USA

Peter McFetridge

USA

Michael McIntosh

USA

Colleen Mcmahan

USA

Andrea Mellitzer

Austria

Rima Menassa

Canada

Torsten Meyer

Germany

Sarah Michel

USA

Allen Miller

USA

Saroj Mishra

India

Christian Mittelholzer

Switzerland

Kimihiko Mizutani

Japan

Mostafa Modarresi

Iran

Jose Moran

Spain

Stefan Müller

Switzerland

Gerhard Müller-Newen

Germany

Fabiola Munarin

Italy

Koji Murai

Japan

Maureen Murphy

USA

Serge Muyldermans

Belgium

Takeharu Nagai

Japan

Akkipeddi Narayana Rao

India

Savi Natarajan

USA

Peter Nicholls

UK

Peter E Nielsen

Denmark

Ken-Ichi Nishijima

Japan

Santosh Noronha

India

Eok-Soo Oh

South Korea

Janet Oldak

USA

Lesley O'Leary

USA

Rosamaria Oliart

Mexico

Takeshi Omasa

Japan

Linda Otten

Netherlands

Ray Owens

UK

Abbasali Palizban

Iran

Hongcheng Pan

China

Christos Panagiotidis

Greece

Viviana Parreno

Argentina

Yoav Peleg

Israel

Suprasanna Penna

India

Helena Persson

Sweden

Nuttaporn Pimpha

Thailand

Perpetua Pinto-Do-Ó

Portugal

Appa Rao Podile

India

László Poppe

Hungary

Marina Porcelli

Italy

Robert Possee

UK

Asmita Prabhune

India

Sriharsa Pradhan

USA

Randall Prather

USA

Archana Pundle

India

Jiayuan Quan

USA

Wim Quax

Netherlands

Manchikatla Rajam

India

Rama Rajaram

India

Aravindan Rajendran

India

Bruce Ramsay

Canada

Priya Ranjan

USA

Jian Rao

USA

Tim Rayner

UK

Heinz Redl

Austria

Peter Richards

Switzerland

Kim Richardson

New Zealand

Maria Manuela Rigano

Italy

Adolfo Rivero

Finland

Gourgopal Roy

USA

Joan Ruderman

USA

Karl Rumbold

South Africa

Holger Russ

USA

Xavier Saelens

Belgium

Rangarajan Sampath

USA

Gerhard Sandmann

Germany

Susanna-Assunta Sansone

UK

Axel Schambach

Germany

Joachim Schiemann

Germany

Stefan Schillberg

Germany

Thomas Schirrmann

Germany

Karen-Beth Scholthof

USA

Bianca Sclavi

France

Richard Scott

New Zealand

Soundarapandian Sekar

India

Weilan Shao

China

Susan Sharfstein

USA

Susan Sharfstein

USA

William Sheffield

Canada

Hiroshi Shimizu

Japan

Tatsuya Shimizu

Japan

Yogesh Shouche

India

Sudhir Singh

India

Giorgio Stanta

Italy

Kathryn Stowell

New Zealand

Wei Wen Su

USA

Seiji Takahashi

Japan

Hideki Tani

Japan

Bill Tawil

USA

Xavier Terrien

France

Roger Thilmony

USA

Robert Thomas

UK

James Thomson

USA

Jim Thorsen

Norway

Oleg Tolmachov

UK

Mario Tosi

France

Jorg Tost

France

Jacques Tremblay

Canada

Maobing Tu

USA

Emily Turner

USA

Ossi Turunen

Finland

Hiroshi Ueda

Japan

Edelmira Valero

Spain

Els Van Damme

Belgium

John Van Der Oost

Netherlands

Leo Van Der Pol

Netherlands

Kristina Vartanian

USA

Vinod Vathipadiekal

USA

Karuppannan Veluthambi

India

Yuhya Wakasa

Japan

Yizao Wan

China

Xiuli Wang

China

Hongjun Wang

USA

Shifeng Wang

USA

Sven Wellmann

Switzerland

Nils Wichmann

Germany

Joerg Wiedenmann

UK

Matthew Wilson

USA

Paul Wingfield

USA

Helen Wise

UK

Alexandre Wohlkonig

Belgium

Tony Wong

Hong Kong

Sook San Wong

USA

Rick Wood

USA

David Wood

USA

Gavin Wright

UK

Florian Wurm

Switzerland

Soichiro Yamada

USA

Guoli Yang

China

Weien Yuan

China

Jianguo Zhang

China

Xing Zhang

USA

Jibin Zhang

China

Qingshun Zhao

China

Xiao-Ling Zhao

China

Changyu Zheng

USA

Zeng-Rong Zhu

China

